# Performance Assessment of Centrifugation-Based Hematocrit Testing in Primary Care Units: Evidence from Seventy-Two Proficiency Testing Datasets over Eight Years

**DOI:** 10.3390/diagnostics16193252

**Published:** 2026-10-08

**Authors:** Watcharinthon Theansun, Kunchit Kongros, Napaporn Apiratmateekul, Ornuma Haonon, Munthana Wadthaisong, Butsabawan Chaireunsung, Gerald J. Kost, Wanvisa Treebuphachatsakul

**Affiliations:** 1 Department of Medical Technology, Faculty of Allied Health Sciences, Naresuan University, Phitsanulok 65000, Thailand; watcharinthont@nu.ac.th (W.T.); kunchitk@nu.ac.th (K.K.); napaporna@nu.ac.th (N.A.); ornumah@nu.ac.th (O.H.); 2 Diagnostic and Wellness Innovation Cluster, Naresuan University, Phitsanulok 65000, Thailand; munthanaw@nu.ac.th (M.W.); geraldkost@gmail.com (G.J.K.); 3 Reference Materials and Innovation Research Unit for Medical Laboratory, Faculty of Allied Health Sciences, Naresuan University, Phitsanulok 65000, Thailand; 4 Department of Cardio-Thoracic Technology, Faculty of Allied Health Sciences, Naresuan University, Phitsanulok 65000, Thailand; 5 We Med Lab Proficiency Testing Center, WE Med Lab Center Co., Ltd., Phitsanulok 65000, Thailand; butsabawanc.wemedlab@gmail.com; 6 Department of Pathology and Laboratory Medicine, POCT.CTR, School of Medicine, University of California, Davis 95616, CA, USA

**Keywords:** hematocrit, quality assurance, point-of-care testing, proficiency testing, performance evaluation, pooled relative standard deviation, anemia screening, anemia monitoring

## Abstract

**Background:** Centrifugation-based hematocrit (Hct) testing remains widely used in decentralized primary care settings where access to automated hematology analyzers may be limited. Appropriate proficiency testing (PT) performance assessment therefore requires consideration of the analytical variability associated with this measurement method. This study evaluated longitudinal PT data to characterize method-specific variability and compare alternative approaches to RSD estimation for centrifugation-based Hct testing. **Methods:** Seventy-two datasets from 32 PT rounds conducted during 2017–2024 were analyzed, with 12 to 310 participants per dataset. PT materials were assessed for homogeneity and stability in accordance with ISO 13528. The relative standard deviation (RSD) was estimated using two approaches: Method A, based on all statistically processed valid results to reflect total interlaboratory variability, and Method B, based on the satisfactory-performance subset (∣*z*∣ ≤ 2), within which robust statistics were recalculated. Temporal trends were evaluated by linear regression using dataset-specific RSD values. Sensitivity analysis evaluated Method B using alternative z-score inclusion thresholds of ∣*z*∣ ≤ 1.5, 2.0, 2.5, and 3.0. Performance classifications in 2024 were compared descriptively. **Results:** All PT materials met predefined homogeneity and stability criteria. The final cumulative pooled relative standard deviation (RSD_pooled_) was 10.81% for Method A and 4.86% for Method B. was 10.81% for Method A and 4.86% for Method B. Method A showed an increasing temporal trend, whereas Method B showed minimal temporal change; however, participant numbers increased substantially over the study period and may have contributed to the Method A trend. Sensitivity analysis showed that Method B was threshold-dependent, with final RSD_pooled_ values of 3.90%, 4.86%, 5.59%, and 6.28% at ∣*z*∣ ≤ 1.5, 2.0, 2.5, and 3.0, respectively. In the 2024 datasets, satisfactory rates ranged from 75.68–99.12% for Method A and 43.15–95.61% for Method B. **Conclusions:** Methods A and B provide complementary information whereas Method B characterizes dispersion among participants meeting a predefined satisfactory-performance criterion. The Method B RSD_pooled_ of 4.86% is specific to inclusion criterion and should not be interpreted as a threshold-independent estimate of analytical imprecision. Further studies using prospectively defined statistical methods and appropriately structured participant-level data are needed to determine its potential utility for PT/EQA performance assessment.

## 1. Introduction

Anemia remains a major global public health problem, particularly in low- and middle-income countries, where its prevalence exceeds 40% among vulnerable populations in Southeast Asia [1,2]. Hematocrit (Hct), defined as the proportion of red blood cells relative to total blood volume, is widely used for anemia screening and monitoring [3]. Although automated hematology analyzers are routinely used in centralized laboratories, centrifugation-based Hct measurement remains relevant in decentralized primary care settings where access to automated hematology analyzers may be constrained by infrastructure, equipment availability, and operational factors [3,4,5,6,7]. In Thailand, this method is routinely used across different levels of care, including 9877 subdistrict health-promoting hospitals (SDHHs) that provide community-level health services [8]. Ensuring reliable analytical performance of centrifugation-based Hct testing is therefore important for maintaining the quality of decentralized testing services.

External quality assessment (EQA), including proficiency testing (PT), provides an objective means of evaluating interlaboratory analytical performance [9]. In PT, the standard deviation for proficiency assessment (σ) is a critical parameter because it determines the allowable dispersion used in z-score calculations and consequently influences participant performance classification [9,10]. For Hct testing, performance specifications are commonly derived from criteria developed primarily for automated hematology systems [11]. However, centrifugation-based Hct measurement differs substantially from automated measurement in analytical principle, operator involvement, equipment, and testing environment. Consequently, performance specifications derived from automated systems may not adequately represent the variability achievable by centrifugation-based Hct testing in decentralized settings.

Longitudinal PT data obtained using homogeneous and stable blood materials provide an opportunity to characterize analytical variability under routine testing conditions. The pooled relative standard deviation (RSD) may be particularly informative because it summarizes variability across multiple PT datasets rather than relying on the dispersion observed in a single PT round. However, the resulting RSD may depend on how participant data are selected for pooling, and different RSD estimates may lead to different z-score-based performance classifications. Long-term evaluation is therefore useful for characterizing the behavior of alternative RSD estimation approaches in centrifugation-based Hct PT.

We hypothesized that longitudinal PT data could be used to characterize long-term variability in centrifugation-based Hct testing and that different approaches to RSD estimation would exhibit different long-term patterns and yield different descriptive patterns of z-score-based performance classification. Accordingly, this study analyzed 8 years of Hct PT data generated using homogeneous and stable blood materials to (1) compare two approaches for estimating RSD, (2) evaluate their long-term trends and stability, (3) characterize their effects on z-score-based performance classification, and (4) assess the potential utility and limitations of the resulting long-term RSD estimates for quality assessment of centrifugation-based Hct testing, particularly in decentralized primary care settings where automated hematology testing is not routinely available.

## 2. Materials and Methods

This study used anonymized proficiency testing program data and laboratory quality management data. No individual patient information or identifiable personal data were collected, accessed, or analyzed. Blood materials used for PT material preparation were obtained from a certified manufacturer of medical devices and medical reference materials operating under established quality management systems. Therefore, ethical approval and informed consent were not required.

Generative artificial intelligence (ChatGPT [GPT-5.5 Thinking]; OpenAI, San Francisco, CA, USA) was used only for language editing and improving manuscript readability. The authors reviewed and verified all content and take full responsibility for the final manuscript.

### 2.1. Study Data and PT Program

Hematocrit (Hct) results obtained by centrifugation were compiled from an 8-year proficiency testing (PT) program conducted between 2017 and 2024. The final analytical dataset consisted of 72 eligible datasets derived from 64 PT materials distributed over 32 PT rounds. The We Med Lab Proficiency Testing Center, accredited as a PT provider under ISO/IEC 17043; Conformity Assessment—General Requirements for the Competence of Proficiency Testing Providers and as a reference material producer under ISO 17034 by the Department of Science Service, Thailand [9,12], was responsible for preparing and distributing PT materials, receiving participant results, and recording PT data.

### 2.2. Homogeneity and Stability Testing of Hct PT Materials

Before distribution, the homogeneity of each batch of Hct PT material was assessed in accordance with ISO 13528 [10]. Ten tubes from each batch were selected by independent random sampling, and duplicate measurements were performed for each tube. Stability testing was also conducted according to ISO 13528 [10]. Four tubes were selected by independent random sampling to evaluate transport stability and end-of-study stability at 3 weeks after the distribution date.

### 2.3. Data Handling and Analytical Subset Selection

**Figure 1:**
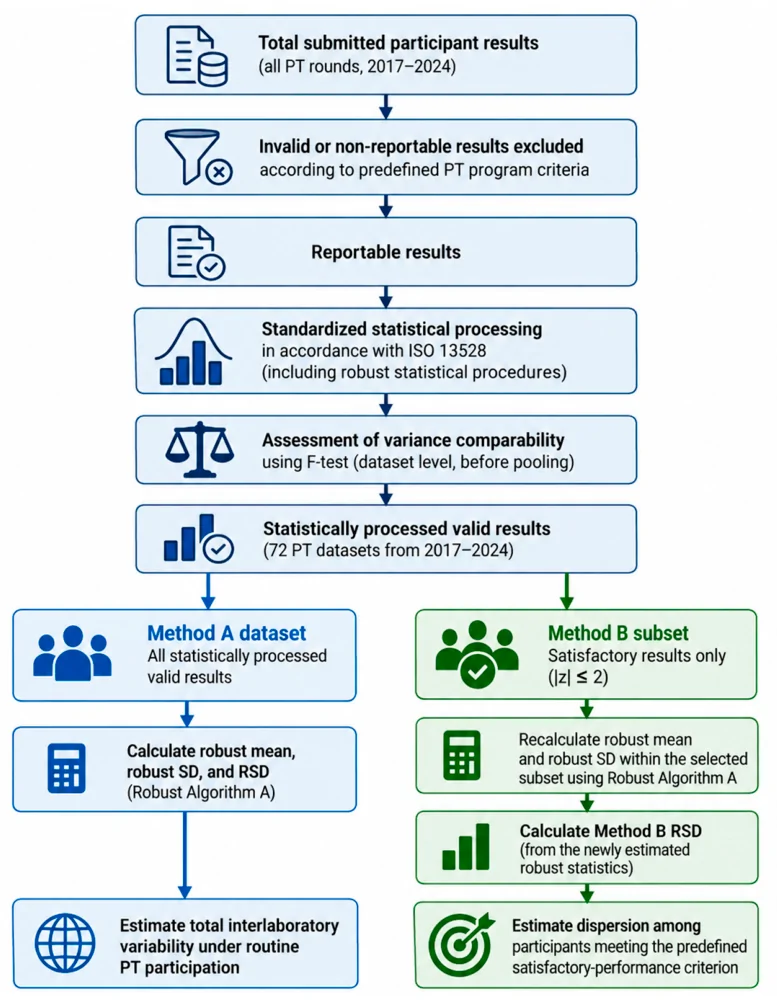
Flow diagram of data handling and analytical subset selection for centrifugation-based hematocrit proficiency testing (PT), 2017–2024. Arrows indicate the sequential data-processing and subset-selection steps. Blue and green represent Method A (all statistically processed valid results) and Method B (results satisfying the predefined criterion, |*z*| ≤ 2), respectively. For Method B, the robust mean and robust SD were recalculated within the selected subset using Robust Algorithm A, and the corresponding RSD was derived from these recalculated statistics. Icons are used for visual illustration only.

The overall data handling workflow is summarized in Figure 1. All submitted participant results were first reviewed for reportability. Invalid or non-reportable results, if present, were excluded according to predefined PT program criteria. The remaining reportable results were then processed using standardized statistical procedures in accordance with ISO 13528. No participant result was manually excluded by the researchers.

After this standardized data-processing step, two analytical approaches were evaluated. Method A included all statistically processed valid participant results and was used to estimate total interlaboratory dispersion under routine PT participation. Method B used the same processed dataset as Method A but restricted the pooled variance estimation to participant results classified as satisfactory (∣*z*∣ ≤ 2), according to the predefined PT performance evaluation procedure for each PT round. After selection of results satisfying ∣*z*∣ ≤ 2, the robust mean and robust SD were recalculated within the selected subset using Robust Algorithm A, and the corresponding Method B RSD was calculated from these newly estimated robust statistics.

### 2.4. RSD and Pooled RSD Calculations

A total of 74 PT datasets were initially available for pooled analysis. Before pooling, the comparability of variances among datasets obtained from PT materials at different Hct concentrations was evaluated using the F-test. Datasets showing no statistically significant difference in variance were considered suitable for pooled RSD estimation. Two datasets did not meet this criterion and were excluded, leaving 72 eligible datasets for subsequent pooled and longitudinal analyses.

The relative standard deviation (RSD) was expressed as a percentage for each dataset, where *n* represented the number of participating laboratories in each PT dataset.

The pooled relative standard deviation (RSD) was calculated according to ISO 5725 [13] using the following equation:

$${\mathrm{RSD}}_{\mathrm{pooled}}=\sqrt{\left(\frac{\left(n_{1}-1\right)\times {\mathrm{RSD}}_{1}^{2}+\left(n_{2}-1\right)\times {\mathrm{RSD}}_{2}^{2}+\ldots \ldots .}{\left(n_{1}-1\right)+\left(n_{2}-1\right)+\ldots }\right)}$$

Long-term RSD was estimated cumulatively across the study period. Beginning with the eligible PT datasets from 2017, datasets from each subsequent year were sequentially added to the pooled calculation. Thus, each annual cumulative RSD value represents the pooled estimate derived from all eligible datasets from 2017 through the corresponding year, and the final 2024 RSD values represent the long-term estimates derived from all eligible PT datasets collected during 2017–2024.

### 2.5. Trend and Stability Analysis of RSD

Temporal trends in dataset-specific RSD values for Methods A and B across the 72 eligible PT datasets were evaluated using linear regression, with PT datasets arranged in chronological order. Regression slopes were expressed as percentage points per PT dataset. Round-specific robust SD values were also plotted to illustrate short-term variation across PT datasets.

### 2.6. Performance Evaluation of Hct Measurement

After each PT distribution round, participating laboratories submitted their Hct results to the PT provider. The assigned value for each material level was determined using the consensus value calculated by Robust Algorithm A, as recommended in ISO 13528 [10]. This procedure yielded the robust mean, robust standard deviation (Robust SD), and standard uncertainty of the assigned value (ux_pt_).

Participant performance was evaluated using z-scores. Results were classified as satisfactory when ∣*z*∣ ≤ 2.0, questionable when 2.0 < ∣*z*∣ < 3.0, and unsatisfactory when ∣*z*∣ ≥ 3.0. The z-score and the standard deviation for proficiency assessment (*σ_pt_*) were calculated using the selected RSD percentage in accordance with ISO 13528 [10].

### 2.7. Comparison of Performance Classification Across RSD Estimation Approaches

For the 2024 performance comparison, participant results were classified as satisfactory when ∣*z*∣ ≤ 2.0 and non-satisfactory when ∣*z*∣ > 2.0. Performance classifications were summarized as counts and percentages for three RSD estimation approaches: Method A-derived RSD_pooled_, Method B-derived RSD_pooled_, and round-specific robust SD.

Because the same participant results were evaluated under all three approaches, the resulting classifications were not independent. In addition, participant-level matched identifiers were not available in the retrospective dataset to support paired or repeated-measures analysis. Therefore, no inferential statistical test was performed to compare classification proportions among the three approaches, and the results were interpreted descriptively.

## 3. Results

### 3.1. Homogeneity and Stability of Hct PT Materials

The homogeneity and stability of the Hct PT materials used throughout the 2017–2024 study period are summarized in Table S1. All materials met the predefined acceptance criteria for homogeneity before distribution and remained acceptable throughout the specified stability period, in accordance with ISO 13528:2022. Although individual materials were classified as either adequate or sufficient depending on the observed between-sample variation and changes during stability assessment, none failed the predefined acceptance criteria. These findings confirm that the PT materials used to generate the longitudinal datasets had acceptable homogeneity and stability, supporting their use for subsequent evaluation of interlaboratory variability and RSD_pooled_.

### 3.2. RSD and Pooled RSD of Centrifugation-Based Hct Testing

The assigned robust mean, robust SD, RSD, and standard uncertainty of the assigned value (u_xpt_) for Hct measurement by centrifugation, using 64 PT materials from 2017 to 2024, are summarized in Table 1. The Method A-derived RSD_pooled_ was 10.81%, whereas the Method B-derived RSD_pooled_ was 4.86%.

Before pooling, variance homogeneity among datasets was assessed using the F-test. Datasets meeting the variance homogeneity criterion (F < F_critical_) were considered suitable for pooled RSD estimation. These findings indicated that the pooled RSD estimates could be calculated using statistically comparable datasets within the predefined analytical framework.

### 3.3. RSD Estimation, Performance Classification, and Temporal Trends

Table 2 compares performance classification across the three PT rounds conducted in 2024 using different approaches for defining σ. With the Method A-derived RSD of 10.81%, satisfactory performance ranged from 75.68% to 99.12% across the six PT datasets. The corresponding satisfactory rates were 43.15–95.61% using the Method B-derived RSD of 4.86% and 86.08–95.61% using the round-specific robust SD. These classification results are presented descriptively because no inferential statistical comparison among the three approaches was performed.

**Figure 2:**
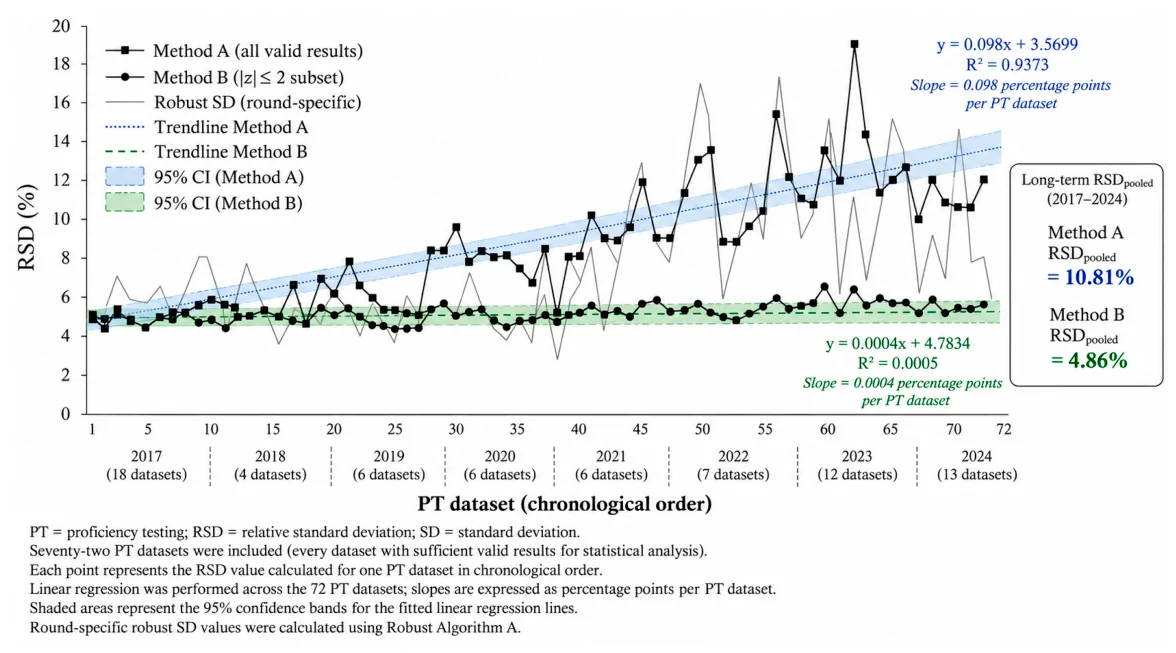
Temporal trends in relative standard deviation (RSD) across 72 chronologically ordered proficiency testing (PT) datasets from 2017 to 2024. Each point represents the RSD calculated for an individual PT dataset. Method A included all statistically processed valid results, whereas Method B included the subset satisfying the predefined satisfactory-performance criterion (|*z*| ≤ 2). Linear regression was performed across the 72 datasets in chronological order, with slopes expressed as percentage points per PT dataset; shaded areas represent the 95% confidence bands for the fitted regression lines. The final cumulative RSD_pooled_ values for 2017–2024 were 10.81% for Method A and 4.86% for Method B. Round-specific robust SD values were calculated using Robust Algorithm A.

Figure 2 shows the temporal trends in RSD across 72 PT datasets arranged chronologically from 2017 to 2024. Each observation represents the RSD calculated for an individual PT dataset. Method A showed a progressive increase over time (slope = 0.098 percentage points per PT dataset; R² = 0.9373), whereas Method B showed minimal temporal change (slope = 0.0004 percentage points per PT dataset; R² = 0.0005). Round-specific robust SD showed greater short-term fluctuations without a consistent long-term trend. Thus, Method B showed less temporal variation than Method A over the study period; however, this estimate remained dependent on the predefined inclusion criterion.

Sensitivity analysis showed that the Method B RSD_pooled_ depended on the z-score inclusion threshold. The final cumulative RSD_pooled_ was 3.90% at ∣*z*∣ ≤ 1.5, 4.86% at ∣*z*∣ ≤ 2.0, 5.59% at ∣*z*∣ ≤ 2.5, and 6.28% at ∣*z*∣ ≤ 3.0 (Table S2). Thus, progressively broader inclusion thresholds generally produced higher estimates of pooled analytical dispersion.

### 3.4. Performance Classification Results Across RSD Estimation Approaches

Across the six PT datasets evaluated in 2024, 1128 participant-result classifications were evaluated under each RSD estimation approach. Method A classified 964 results (85.46%) as satisfactory, compared with 740 (65.60%) for Method B and 1010 (89.54%) for the round-specific robust SD approach. Across individual datasets, satisfactory rates ranged from 75.68% to 99.12% for Method A, 43.15% to 95.61% for Method B, and 86.08% to 95.61% for the round-specific robust SD approach (Table 3). These results are presented as descriptive comparisons; no inferential statistical test was performed because the classifications generated by the three approaches were based on the same participant results and could not be treated as independent observations.

## 4. Discussion

This study demonstrated that the RSD_pooled_ derived from Method B stabilized at approximately 4.86% when calculated across multiple PT cycles over an 8-year period. The use of long-term pooled variability across repeated PT rounds may reduce the influence of round-specific fluctuations commonly observed in EQA/PT programs and provide a more stable estimate of dispersion over time. However, the sensitivity analysis demonstrated that the Method B estimate was dependent on the predefined z-score inclusion threshold. Therefore, the value of 4.86% should be interpreted specifically as the dispersion observed among participants meeting the predefined satisfactory-performance criterion (∣*z*∣ ≤ 2.0), rather than as a threshold-independent estimate of analytical imprecision. Nevertheless, periodic recalculation of RSD_pooled_ remains important to ensure that performance benchmarks continue to reflect current practice in primary care settings, such as SDHHs. This is particularly relevant in Thailand, where the number of SDHHs is estimated to be approximately 10 times greater than the number of hospital-based laboratories [9], creating a large decentralized testing network with varying levels of operator experience, equipment management, and resource availability.

Our findings indicate that the method used to estimate RSD substantially influenced performance classification. Because Methods A and B used the same standardized data-processing procedures before pooled RSD estimation, the observed differences were primarily attributable to the analytical subset used for RSD_pooled_ calculation. Method A included all statistically processed valid participant results and therefore reflected total interlaboratory dispersion under routine PT participation. In contrast, Method B was derived from the satisfactory subset of the same processed dataset and characterized dispersion among participants meeting the predefined satisfactory-performance criterion. Selection dependency is therefore inherent in Method B. This interpretation was supported by the sensitivity analysis, in which the final cumulative RSD_pooled_ was 3.90% at ∣*z*∣ ≤ 1.5, 4.86% at ∣*z*∣ ≤ 2.0, 5.59% at ∣*z*∣ ≤ 2.5, and 6.28% at ∣*z*∣ ≤ 3.0. Thus, progressively broader inclusion thresholds generally resulted in higher estimates of pooled dispersion. These findings indicate that the 4.86% estimate is specific to the predefined ∣*z*∣ ≤ 2.0 criterion and should not be regarded as a threshold-independent measure of intrinsic analytical imprecision. Rather, it represents a criterion-dependent estimate that may be useful as a candidate method-specific benchmark for quality improvement. The selection of σ_pt_ is a critical determinant of performance classification in PT schemes, as ISO 13528 emphasizes that the dispersion parameter directly influences z-score interpretation and participant grading [10,14]. Inappropriate estimation of σ_pt_ may therefore result in systematic over- or under-classification of laboratory performance [15].

The progressive increase in Method A-derived RSD may reflect expansion of PT participation to a broader and more heterogeneous group of decentralized testing sites over time. Early PT rounds included fewer participants, whereas later rounds included substantially larger numbers of primary care and decentralized sites with greater variation in operator experience, equipment maintenance, capillary tube handling, centrifugation conditions, reading technique, and documentation practices. Therefore, the increase observed for Method A should not be interpreted solely as deterioration of analytical performance; changes in participant number and network composition over time may also have contributed to the observed increase in interlaboratory variability. This finding supports the need to monitor total interlaboratory dispersion using Method A while using Method B to characterize dispersion within a predefined satisfactory-performance subset.

Comparing the RSD_pooled_ values from Methods A and B with CLIA performance requirements revealed important methodological differences. Using the CLIA 2019 allowable limit of ±6.00% [16], which was primarily established for automated hematology analyzers, the Method A-derived RSD_pooled_ exceeded this threshold, whereas the Method B-derived RSD_pooled_ remained below it. The updated CLIA 2024 criteria [11] further tightened the allowable Hct limit to ±4.00%, reflecting technological advances in automated hematology systems. However, performance specifications should consider analytical principles, measurement uncertainty, intended clinical use, and testing environment rather than applying universal criteria across heterogeneous methodologies [17,18]. Consequently, direct application of CLIA performance limits to centrifugation-based Hct testing may be inappropriate, particularly in decentralized or point-of-care testing environments where analytical variability differs from that of central laboratory systems [19].

The findings of this study have practical implications for EQA/PT providers responsible for centrifugation-based Hct schemes. A method-specific RSD_pooled_ derived from longitudinal field data may help PT providers design performance evaluation models that better reflect the analytical characteristics and implementation context of centrifugation-based Hct testing. However, the Method B-derived RSD_pooled_ of approximately 4.86% should not be interpreted as an immediate or absolute pass/fail criterion for all primary care sites. Because this estimate is specific to the predefined ∣*z*∣ ≤ 2.0 inclusion criterion, it may instead serve as a candidate criterion-dependent, method-specific benchmark for quality improvement in decentralized hematology services, subject to prospective validation.

For practical implementation, an allowable total error of 6.00% may provide a pragmatic initial reference for SDHHs undergoing quality improvement of centrifugation-based Hct testing. As laboratory competency, operator skill, internal quality control (IQC) practice, equipment management, and PT/EQA performance improve, the Method B-derived RSD_pooled_ may provide additional information for considering more stringent performance targets. However, because the 4.86% estimate is criterion-dependent, its adoption as a formal performance specification or σ_pt_ should require prospective validation. This staged approach may be more appropriate for decentralized testing networks than immediate application of a highly stringent criterion.

Our findings are consistent with previous studies of centrifugation-based Hct testing in decentralized settings. Apiratmateekul et al. [20] demonstrated satisfactory performance of centrifugation-based Hct testing in 274 SDHHs, reporting an overall satisfactory rate of 91.30% using an RSD_pooled_ value of 4.95%. Similar findings were also reported by Sakaew et al. [21]. These results support the concept that performance criteria derived from empirical field data may better reflect the analytical characteristics of centrifugation-based Hct testing in decentralized healthcare settings than criteria derived primarily from automated central laboratory systems [22]. Nevertheless, the present sensitivity analysis further demonstrates that estimates derived from selected participant subsets are influenced by the inclusion criterion and should therefore be interpreted within the context of the criterion used to derive them.

The proposed Method B-derived RSD_pooled_ of approximately 4.86% may have several practical applications for centrifugation-based Hct testing in primary care units. As a candidate criterion-dependent benchmark, it may support PT/EQA performance evaluation, planning of IQC procedures, method verification, staff competency assessment, and longitudinal system monitoring. Importantly, it should be used as a quality-improvement reference rather than as an independently validated pass/fail limit. Continued monitoring using Method A remains necessary to capture total interlaboratory variability and identify changes in performance across the broader decentralized testing network.

Reliable Hct results are important for reducing the risk of misclassification, inappropriate referral, and delayed treatment, particularly among vulnerable populations such as children, pregnant women, and older adults. Because SDHHs provide frontline Hct testing nationwide, the use of method-appropriate and prospectively validated performance criteria may strengthen analytical quality and support clinical decision-making across Thailand’s primary healthcare system.

Limitations, this study has several limitations. First, the analysis was based on retrospective PT data from centrifugation-based Hct testing in Thailand; therefore, the findings may not be directly generalizable to other healthcare systems or testing environments. Second, although the longitudinal dataset covered 8 years and multiple PT cycles, changes in the number and characteristics of participating sites over time may have contributed to the observed interlaboratory variability. Third, Method B was derived from the satisfactory subset (∣z∣ ≤ 2.0) of the statistically processed data and is therefore inherently selection- and threshold-dependent. Sensitivity analysis confirmed this dependency, with the final cumulative RSD_pooled_ ranging from 3.90% to 6.28% across the evaluated inclusion thresholds. Accordingly, the Method B-derived RSD_pooled_ of 4.86% should be interpreted specifically as an empirical estimate of dispersion among participants meeting the predefined ∣z∣ ≤ 2.0 satisfactory-performance criterion, rather than as a universal or threshold-independent estimate of analytical imprecision. Further prospective evaluation across independent PT programs and decentralized testing networks is warranted before its adoption as a formal σ_pt_ or PT/EQA performance specification.

Taken together, Methods A and B should be regarded as complementary rather than competing approaches. Method A reflects total interlaboratory variability under routine PT participation, whereas Method B characterizes dispersion among participants meeting a predefined satisfactory-performance criterion. The Method B-derived RSD_pooled_ of approximately 4.86% should therefore be interpreted within the context of the inclusion criterion used to derive it. Further studies using prospectively defined statistical methods and appropriately structured participant-level data are needed to determine its potential utility for PT/EQA performance assessment.

## 5. Conclusions

The Method B-derived RSDpooled of approximately 4.86% represents an empirically derived, criterion-dependent estimate of dispersion among participants meeting the predefined satisfactory-performance criterion (|*z*| ≤ 2.0) for centrifugation-based Hct testing. Method A and Method B provide complementary descriptive information on total interlaboratory variability and dispersion within the predefined satisfactory-performance subset, respectively. The 4.86% estimate should therefore be interpreted within the context of the inclusion criterion used to derive it and should not be considered a validated performance specification. Further studies using prospectively defined statistical methods and appropriately structured participant-level data are needed to determine its potential utility for PT/EQA performance assessment.

## Figures and Tables

**Table 1:** Assigned robust mean, robust standard deviation (Robust SD), and relative standard deviation (RSD) of the proficiency testing program for hematocrit measurement by centrifugation, from 2017 to 2024.

Year	PT Material Code	Number of Participants	Method A			Method B		
			Robust Mean x* (%)	Robust SD s* (%)	RSD (%)	Robust Mean x* (%)	Robust SD s* (%)	RSD (%)
2017	60-803 (L)	27	24.10	1.70	7.05	23.89	1.49	6.22
	60-804 (H)	27	42.00	2.60	6.19	41.75	2.29	5.49
	60-805 (M)	30	37.50	1.90	5.07	37.33	1.68	4.51
	60-806 (H)	30	42.80	2.00	4.67	42.86	1.62	3.78
	60-807 (H)	31	47.77	2.08	4.35	47.89	1.64	3.43
	60-808 (L)	33	25.27	1.34	5.30	25.27	0.87	3.46
	60-809 (L)	33	22.00	1.30	5.91	22.32	1.19	5.35
	60-810 (M)	32	39.00	2.10	5.38	39.16	1.92	4.89
	60-813 (H)	31	45.00	3.34	7.42	45.48	2.61	5.75
	60-814 (L)	31	27.00	1.97	7.30	27.15	2.41	8.49
	60-815 (H)	16	40.92	2.83	7.91	40.32	2.42	6.00
	60-816 (H)	16	44.17	2.79	6.32	43.04	2.19	5.09
	60-811 (L)	12	27.75	1.61	7.09	22.75	1.42	6.25
	60-812 (H)	12	38.84	2.17	5.59	38.55	1.69	4.40
	60-817 (H)	12	49.36	2.01	4.06	49.33	1.83	3.70
	60-818 (L)	12	28.82	1.40	4.86	28.40	0.84	2.97
	60-219 (H)	12	47.60	1.59	4.34	47.58	1.44	3.03
	60-220 (M)	12	37.00	1.37	3.70	37.00	1.21	3.26
	RSD_pooled_ (%)				5.85	RSD_pooled_ (%)		5.22
2018	61-203 (H)	55	47.08	2.02	4.29	47.33	1.80	3.81
	61-204 (L)	55	27.39	1.51	5.51	27.54	1.23	4.47
	61-205 (M)	55	39.43	3.22	8.27	38.83	2.36	6.08
	61-206 (H)	55	48.72	2.66	5.46	49.22	1.97	4.01
	RSD_pooled_ (%)				5.91	RSD_pooled_ (%)		5.04
2019	62-297 (H)	62	47.39	1.77	3.73	47.42	1.45	3.06
	62-298 (M)	62	35.49	1.16	3.24	35.46	0.78	2.20
	62-202 (H)	72	48.22	2.07	4.29	48.17	1.81	3.76
	62-244 (L)	63	28.00	1.19	4.03	27.98	1.07	3.82
	62-245 (M)	63	46.69	1.88	3.45	46.91	1.58	3.37
	62-243 (L)	63	36.55	1.26	4.35	36.63	1.15	3.15
	RSD_pooled_ (%)				5.22	RSD_pooled_ (%)		4.45
2020	63-266 (M)	185	38.71	3.36	8.68	39.17	1.81	4.61
	63-267 (L)	185	26.35	2.41	9.15	26.22	1.08	4.12
	63-204 (L)	307	27.23	2.70	9.92	27.02	1.25	4.64
	63-205 (H)	306	45.44	4.22	9.29	46.08	1.93	4.18
	63-249 (M)	85	37.03	3.22	8.70	37.36	2.31	6.19
	63-250 (H)	85	46.75	2.15	4.60	46.75	1.54	3.30
	RSD_pooled_ (%)				7.18	RSD_pooled_ (%)		4.47
2021	64-254 (M)	83	40.45	1.28	3.26	40.32	1.15	2.86
	64-255 (H)	82	49.88	1.57	3.23	49.86	1.55	3.12
	64-237 (H)	264	47.91	4.04	8.40	48.88	2.03	4.16
	64-238 (L)	264	30.00	2.65	8.80	30.23	1.61	5.32
	64-201 (L)	92	29.33	0.94	3.20	29.32	0.98	3.33
	64-202 (M)	92	37.35	1.22	3.30	37.37	1.20	3.22
	RSD_pooled_ (%)				7.07	RSD_pooled_ (%)		4.38
2022	65-244 (M)	257	34.81	3.64	10.50	34.95	2.37	6.57
	65-255 (H)	258	45.21	4.36	9.60	45.79	2.83	6.18
	65-232 (L)	157	27.51	2.44	8.90	27.59	1.77	6.42
	65-222 (H)	158	48.98	3.48	7.10	49.71	2.54	5.11
	65-225 (L)	234	26.19	2.92	11.10	26.34	1.52	5.75
	65-280 (L)	57	26.32	2.49	9.50	26.49	1.52	5.73
	65-290 (H)	57	44.09	3.99	9.00	44.31	3.39	7.66
	RSD_pooled_ (%)				8.10	RSD_pooled_ (%)		5.00
2023	66-218 (M)	106	36.42	2.57	7.10	36.04	1.90	5.29
	66-228 (H)	106	43.93	2.76	6.30	43.49	1.80	4.14
	66-218 (M)	293	35.20	5.69	16.20	35.14	1.70	4.84
	66-228 (H)	292	42.56	7.67	18.00	42.26	2.21	5.23
	66-251 (H)	92	42.35	2.42	5.70	42.63	1.78	4.18
	66-252 (L)	92	27.56	1.70	6.20	27.65	1.29	4.65
	66-236 (L)	107	26.22	1.01	3.90	26.22	0.93	3.56
	66-246 (M)	107	35.75	1.10	4.40	35.81	1.05	2.92
	66-236 (L)	254	25.01	3.74	15.00	34.38	2.06	6.00
	66-246 (M)	252	34.15	3.92	11.20	44.60	2.32	5.21
	66-234 (H)	17	43.96	3.21	7.70	24.69	1.08	4.37
	66-243 (L)	20	24.34	1.83	7.50	26.07	1.08	4.13
	RSD_pooled_ (%)				9.78	RSD_pooled_ (%)		4.96
2024	67-B77 (M)	114	33.14	1.89	5.70	33.06	1.61	4.89
	67-B76 (L)	283	24.95	4.48	18.00	25.26	1.20	4.73
	67-B77 (M)	279	30.91	5.88	19.00	31.03	1.69	5.46
	67-B79 (H)	119	42.83	1.57	5.70	42.83	1.46	3.42
	67-B89 (M)	119	33.45	1.55	4.60	33.47	1.32	3.93
	67-B79 (H)	310	31.22	4.54	14.50	39.84	1.90	4.77
	67-B89 (M)	310	39.00	5.72	14.70	31.91	1.51	4.37
	67-B38 (L)	79	24.49	1.12	4.60	24.51	0.93	3.77
	67-B39 (H)	79	44.13	1.40	4.20	44.10	1.37	3.11
	67-B38 (L)	36	22.81	2.37	10.40	23.43	1.43	6.08
	67-B39 (H)	36	41.52	2.66	6.40	41.84	1.90	4.55
	67-B35 (M)	15	36.00	0.86	2.41	36.00	0.76	2.10
	67-B45 (L)	15	25.47	1.12	4.40	25.47	0.99	3.89
	RSD_pooled_ (%)				10.81	RSD_pooled_ (%)		4.86

Note: x*, robust mean; s*, robust standard deviation; PT, proficiency testing; SD, standard deviation; RSD, relative standard deviation; L, low-level PT materials; M, medium-level PT materials; H, high-level PT materials.

**Table 2:** Performance classification of centrifugation-based hematocrit testing using z-scores calculated from different RSD estimation approaches (PT datasets in 2024).

Source of % RSD	1st Round (Medium Level n = 293, High Level n = 292)					2nd Round (Low Level n = 114, Medium Level n = 114)					3rd Round (Low Level n = 157, High Level n = 158)				
	PT Item	σ_pt_ (RSD)	Robust Mean (%)	Performance n (%)		PT Item	σ_pt_ (RSD)	Robust Mean (%)	Performance n (%)		PT Item	σ_pt_ (RSD)	Robust Mean (%)	Performance n (%)	
				S	U				S	U				S	U
Method A RSD_pooled_ of parameter values for all data from 2017–2024	1 Medium	3.81 (10.81)	35.20	230 (78.50)	63 (21.50)	2 Low	2.83 (10.81)	26.14	113 (99.12)	1 (0.88)	3 Low	2.97 (10.81)	27.51	143 (91.08)	14 (8.92)
	1 High	4.60 (10.81)	42.56	221 (75.68)	71 (24.31)	2 Medium	3.58 (10.81)	33.14	113 (99.12)	1 (0.88)	3 High	5.29 (10.81)	48.98	144 (91.14)	13 (8.86)
Method B RSD_pooled_ derived from satisfactory results (|*z*| ≤ 2)	1 Medium	1.71 (4.86)	35.20	144 (49.15)	149 (50.58)	2 Low	1.27 (4.86)	26.14	109 (95.61)	5 (4.39)	3 Low	1.34 (4.86)	27.51	124 (78.98)	33 (21.02)
	1 High	2.07 (4.86)	42.56	126 (43.15)	166 (56.85)	2 Medium	1.61 (4.86)	33.14	109 (95.61)	5 (4.39)	3 High	2.38 (4.86)	48.98	128 (81.01)	30 (18.99)
The Robust SD from the data of that round	1 Medium	5.69 (16.20)	35.20	262 (89.42)	31 (10.58)	2 Low	1.26 (10.50)	26.14	109 (95.61)	5 (4.39)	3 Low	2.44 (8.90)	27.51	140 (89.17)	17 (10.83)
	1 High	7.67 (18.00)	42.56	254 (86.99)	38 (13.01)	2 Medium	1.89 (9.60)	33.14	109 (95.61)	5 (4.39)	3 High	3.48 (7.10)	48.98	136 (86.08)	22 (13.92)

Note: Hct, hematocrit (packed red blood cell volume); S, satisfactory (|*z*| ≤ 2.0); U, unsatisfactory (|*z*| > 2.0); σ_pt_, standard deviations of the set values; Low, 25–30%; Medium, 30–40%; High, 40–50%; PT, proficiency testing; RSD, relative standard deviation.

**Table 3:** Descriptive summary of performance classifications using different RSD estimation approaches across six PT datasets evaluated in 2024.

RSD Estimation Approach	Satisfactory, n (%)	Unsatisfactory, n (%)	Range of Satisfactory Performance Across Datasets (%)
Method A: cumulative RSD_pooled_ = 10.81%	964 (85.46)	164 (14.54)	75.68–99.12
Method B: cumulative RSD_pooled_ = 4.86%	740 (65.60)	388 (34.40)	43.15–95.61
Round-specific robust SD	1010 (89.54)	118 (10.46)	86.08–95.61

Note: Satisfactory performance was defined as ∣*z*∣ ≤ 2.0, and unsatisfactory performance was defined as ∣*z*∣ > 2.0. Counts represent participant-result classifications across the six PT datasets evaluated in 2024 and should not be interpreted as the number of unique participating facilities. Comparisons among approaches are descriptive; no inferential statistical test was performed because the same participant results were classified under all three approaches.

## Data Availability

Data is available upon request.

## Supplementary Materials

The following supporting information can be downloaded at: https://www.mdpi.com/article/10.3390/diagnostics16193252/s1, Table S1: Homogeneity and stability data of the proficiency testing program for hematocrit measurement by centrifugation from 2017 to 2024; Table S2: Sensitivity analysis of pooled relative standard deviation (RSD_pooled_) for Method B using alternative z-score inclusion thresholds.

## Abbreviations

The following abbreviations are used in this manuscript:

Hct	Hematocrit
PT	Proficiency Testing
EQA	External Quality Assessment
IQC	Internal Quality Control
SDHH	Subdistrict Health-Promoting Hospital
RSD	Relative Standard Deviation
RSD_pooled_	Pooled Relative Standard Deviation
*σ_pt_*	Standard Deviation for Proficiency Assessment
CLIA	Clinical Laboratory Improvement Amendments
PCU	Primary Care Unit
