# Supplementary material for: Performance Assessment of Centrifugation-Based Hematocrit Testing in Primary Care Units: Evidence from Seventy-Two Proficiency Testing Datasets over Eight Years
Source: Diagnostics (Basel). 2026 Oct 8;16(19):3252. doi: 10.3390/diagnostics16193252 (PMC13663924; doi:10.3390/diagnostics16193252)
Supplement: Supplementary file 1 [file diagnostics-16-03252-s001.zip › diagnostics-4531514-supplementary.pdf]

Table S1. Homogeneity and stability data of the proficiency testing program for hematocrit measurement by centrifugation from 2017 to 2024.

Year	PT material Code	Homogeneity				Stability			
		Mean	S_s	0.3 σ_{pt}	Interpretation	Mean	$\lvert y_1 - y_2 \rvert$	0.3 σ_{pt}	Interpretation
2017	60-803 (L)	26.00	0.00	1.17	Adequate	26.40	0.40	1.70	Adequate
	60-804 (H)	44.95	1.23	2.02	Adequate	45.90	0.95	1.70	Adequate
	60-805 (M)	38.40	0.00	0.23	Adequate	38.80	0.40	0.59	Adequate
	60-806 (H)	44.80	0.91	0.68	Sufficient	45.20	0.40	0.59	Adequate
	60-807 (H)	50.45	0.89	0.77	Sufficient	49.80	0.65	0.77	Adequate
	60-808 (L)	27.80	0.54	0.77	Adequate	27.10	0.70	0.77	Adequate
	60-809 (L)	22.70	0.17	1.08	Adequate	23.60	0.90	1.04	Adequate
	60-810 (M)	40.05	0.67	1.08	Adequate	40.10	0.05	1.04	Adequate
	60-813 (H)	46.00	0.57	1.08	Adequate	47.15	1.15	4.02	Adequate
	60-814 (L)	27.00	0.26	1.08	Adequate	27.10	0.10	4.02	Adequate
	60-815 (H)	38.35	0.33	0.64	Adequate	38.60	0.25	0.69	Adequate
	60-816 (H)	41.95	0.36	0.70	Adequate	42.20	0.25	0.69	Adequate
	60-811 (L)	22.70	0.17	4.02	Adequate	23.60	0.90	4.02	Adequate
	60-812 (H)	40.05	0.68	4.02	Adequate	40.10	0.05	4.02	Adequate
	60-817 (H)	51.11	2.55	0.89	Sufficient	50.50	0.61	0.55	Sufficient
	60-818 (L)	29.56	2.07	0.46	Sufficient	30.00	0.44	0.55	Adequate
	60-219 (H)	48.00	0.00	0.77	Adequate	48.50	0.50	0.77	Adequate
	60-220 (M)	38.00	0.00	0.61	Adequate	38.50	0.50	0.61	Adequate
2018	61-203 (H)	48.00	0.00	0.77	Adequate	48.50	0.50	0.77	Adequate
	61-204 (L)	30.00	0.00	0.48	Adequate	30.40	0.40	0.48	Adequate
	61-205 (M)	41.60	0.70	0.63	Sufficient	41.35	0.25	0.64	Adequate
	61-206 (H)	50.75	0.53	0.78	Adequate	51.21	0.46	0.80	Adequate
2019	62-297 (H)	48.90	0.47	0.76	Adequate	48.90	0.00	0.88	Adequate
	62-298 (M)	36.55	0.11	0.56	Adequate	37.00	0.45	0.66	Adequate
	62-202 (H)	49.70	0.59	0.74	Adequate	50.40	0.70	0.74	Adequate
	62-244 (L)	28.20	0.54	0.42	Sufficient	28.20	0.00	0.42	Adequate
	62-245 (M)	47.35	0.00	0.70	Adequate	45.50	1.85	0.70	Sufficient
	62-243 (H)	37.80	0.63	0.56	Sufficient	35.75	1.47	0.56	Sufficient
2020	63-266 (M)	40.70	0.36	0.60	Adequate	39.50	1.20	0.59	Sufficient
	63-267 (L)	28.30	0.48	0.42	Sufficient	27.75	0.55	0.41	Sufficient
	63-204 (L)	27.00	0.00	0.38	Adequate	27.27	0.27	0.67	Adequate
	63-205 (H)	47.90	0.32	0.68	Adequate	47.90	0.00	0.38	Adequate
	63-249 (M)	38.70	0.48	0.55	Adequate	39.00	0.30	0.55	Adequate
	63-250 (H)	49.85	0.00	0.70	Adequate	49.00	0.85	0.69	Sufficient
2021	64-254 (M)	39.65	0.00	0.55	Adequate	39.80	0.15	0.55	Adequate
	64-255 (H)	48.70	0.00	0.68	Adequate	48.90	0.20	0.67	Adequate
	64-237 (H)	50.40	0.52	0.76	Adequate	51.50	1.10	0.77	Sufficient
	64-238 (L)	31.80	0.72	0.48	Sufficient	32.00	0.20	0.48	Adequate
	64-201 (L)	29.30	0.48	0.44	Sufficient	29.25	0.05	0.44	Adequate
	64-202 (M)	37.45	0.11	0.56	Adequate	39.25	1.80	0.59	Sufficient
	65-244 (M)	38.20	0.27	0.80	Adequate	38.00	0.20	0.80	Adequate
	65-255 (H)	50.20	0.42	1.05	Adequate	50.75	0.55	1.07	Adequate
	65-232 (L)	28.45	0.47	0.60	Adequate	29.75	1.30	2.60	Sufficient
	65-222 (H)	52.70	0.48	1.11	Adequate	55.50	2.80	1.17	Sufficient

Year	PT material Code	Homogeneity				Stability			
		Mean	S_s	0.3 σ_{pt}	Interpretation	Mean	$\lvert y_1 - y_2 \rvert$	0.3 σ_{pt}	Interpretation
2022	65-225 (L)	28.20	0.42	0.59	Adequate	28.00	0.20	0.59	Adequate
	65-226 (M)	40.50	0.53	2.10	Adequate	40.75	0.25	0.86	Adequate
	65-280 (L)	29.00	0.00	0.61	Adequate	28.25	0.75	0.59	Sufficient
	65-290 (H)	49.80	0.42	1.05	Adequate	48.25	1.55	1.01	Sufficient
2023	66-218 (M)	37.30	0.48	0.56	Adequate	37.50	0.20	0.56	Adequate
	66-228 (H)	47.05	0.67	0.71	Adequate	47.00	0.05	0.71	Adequate
	66-251 (H)	45.20	0.42	0.68	Adequate	45.50	0.30	0.68	Adequate
	66-252 (L)	29.20	0.42	0.44	Adequate	29.00	0.20	0.44	Adequate
	66-236 (L)	27.60	0.52	0.41	Sufficient	26.75	0.85	0.40	Sufficient
	66-246 (M)	37.45	0.58	0.56	Sufficient	37.00	0.45	0.56	Adequate
	66-234 (H)	46.50	0.53	0.70	Adequate	45.25	1.25	0.68	Sufficient
	66-243 (L)	26.00	0.00	0.39	Adequate	26.00	0.00	0.39	Adequate
2024	67-B76 (L)	27.00	0.00	0.39	Adequate	26.75	0.25	0.39	Adequate
	67-B77 (M)	34.80	0.42	0.49	Adequate	34.25	0.55	0.49	Sufficient
	67-B79 (H)	45.20	0.42	0.50	Adequate	44.25	0.95	0.50	Sufficient
	67-B89 (M)	35.55	0.47	0.64	Adequate	35.00	0.55	0.64	Sufficient
	67-B38 (L)	25.00	0.00	0.36	Adequate	25.00	0.00	0.37	Adequate
	67-B39 (H)	43.80	0.42	0.66	Adequate	44.75	0.95	0.66	Sufficient
	67-B35 (L)	26.20	0.42	0.39	Sufficient	26.25	0.05	0.39	Adequate
	67-B45 (M)	38.20	0.42	0.57	Adequate	37.25	0.95	0.55	Sufficient

Note: S_s, between-sample standard deviation; σ_{pt}, standard deviation for proficiency assessment; y_1, mean value obtained from the homogeneity assessment; y_2, mean value obtained from the stability assessment at the specified storage time; PT, proficiency testing. The acceptance criterion was evaluated against $0.3\sigma_{pt}$in accordance with ISO 13528:2022. The σ_{pt}value was dataset-specific and derived from the robust standard deviation obtained from statistically processed participant results for the corresponding PT dataset.

Adequate: Adequate for the intended purpose indicates that the observed homogeneity or stability met the predefined acceptance criterion and was considered appropriate for use in proficiency testing.

Sufficient: Sufficient for the intended purpose indicates that, although the predefined numerical criterion was not fully met, the observed degree of heterogeneity or instability was considered sufficiently small that it was not expected to adversely affect the evaluation of participant performance, in accordance with ISO 13528:2022, Clause 6.1.1.

Table S2. Sensitivity analysis of pooled relative standard deviation (RSD_{pooled}) for Method B using alternative z-score inclusion thresholds.

Year	|z| ≤ 1.5 RSDpooled (%)	|z| ≤ 2.0 RSDpooled (%)	|z| ≤ 2.5 RSDpooled (%)	|z| ≤ 3.0 RSDpooled (%)
2017	4.48	5.22	5.24	5.10
2018	4.32	5.04	5.22	5.53
2019	3.83	4.45	4.57	4.85
2020	3.73	4.47	4.85	5.40
2021	3.62	4.38	4.82	5.28
2022	4.05	5.00	5.52	6.11
2023	3.93	4.96	5.60	6.34
**2024**	**3.90**	**4.86**	**5.59**	**6.28**

Note: RSD_{pooled} values were recalculated for Method B using alternative z-score inclusion thresholds of $|z| \leq 1.5$, $|z| \leq 2.0$, $|z| \leq 2.5$, and $|z| \leq 3.0$. The $|z| \leq 2.0$ threshold corresponds to the predefined satisfactory-performance criterion used in the primary analysis. Broader inclusion thresholds generally resulted in higher pooled RSD estimates, indicating threshold dependency of the Method B estimate.
